# A novel post-fire method to estimate individual tree crown scorch height and volume using simple RPAS-derived data

**DOI:** 10.1186/s42408-023-00174-7

**Published:** 2023-03-23

**Authors:** Jeremy Arkin, Nicholas C. Coops, Lori D. Daniels, Andrew Plowright

**Affiliations:** 1 Integrated Remote Sensing Studio, Department of Forest Resources Management, University of British Columbia, Vancouver, BC V6T 1Z4 Canada; 2 Tree Ring Lab, Department of Forest and Conservation Sciences, University of British Columbia, Vancouver, BC V6T 1Z4 Canada; 3 Natural Resources Canada, Ottawa, ON Canada

**Keywords:** RPAS, DAP, Wildfire, Severity, Individual tree, Scorch height

## Abstract

**Background:**

An accurate understanding of wildfire impacts is critical to the success of any post-fire management framework. Fire severity maps are typically created from satellite-derived imagery that are capable of mapping fires across large spatial extents, but cannot detect damage to individual trees. In recent years, higher resolution fire severity maps have been created from orthomosaics collected from remotely piloted aerial systems (RPAS). Digital aerial photogrammetric (DAP) point clouds can be derived from these same systems, allowing for spectral and structural features to be collected concurrently. In this note, a methodology was developed to analyze fire impacts within individual trees using these two synergistic data types. The novel methodology presented here uses RPAS-acquired orthomosaics to classify trees based on a binary presence of fire damage. Crown scorch heights and volumes are then extracted from fire-damaged trees using RPAS-acquired DAP point clouds. Such an analysis allows for crown scorch heights and volumes to be estimated across much broader spatial scales than is possible from field data.

**Results:**

There was a distinct difference in the spectral values for burned and unburned trees, which allowed the developed methodology to correctly classify 92.1% of trees as either burned or unburned. Following a correct classification, the crown scorch heights of burned trees were extracted at high accuracies that when regressed against field-measured heights yielded a slope of 0.85, an R-squared value of 0.78, and an RMSE value of 2.2 m. When converted to crown volume scorched, 83.3% of the DAP-derived values were within ± 10% of field-measured values.

**Conclusion:**

This research presents a novel post-fire methodology that utilizes cost-effective RPAS-acquired data to accurately characterize individual tree-level fire severity through an estimation of crown scorch heights and volumes. Though the results were favorable, improvements can be made. Specifically, through the addition of processing steps that would remove shadows and better calibrate the spectral data used in this study. Additionally, the utility of this approach would be made more apparent through a detailed cost analysis comparing these methods with more conventional field-based approaches.

## Resumen

**Antecedentes:**

Un preciso entendimiento de los impactos de los incendios de vegetación es crítico para el éxito de cualquier esquema de manejo posterior. Los mapas de severidad del fuego son creados típicamente desde imágenes derivadas de satélites, siendo capaces de mapear incendios de extensiones espaciales muy grandes, pero no pueden detectar daños en árboles individuales. En años recientes, los mapas de severidad de más alta resolución han sido creados desde ortomosaicos colectados desde sistemas aéreos pilotados de manera remota (RPAS). Los puntos de nubes fotogramétricas digitales pueden ser derivados de esos mismos sistemas, permitiendo que las características espectrales y estructurales sean colectados de manera conjunta. En esta nota, fue desarrollada una metodología para analizar los impactos de los incendios en árboles individuales usando dos tipos de datos sinérgicos. La metodología novedosa aquí presentada usa ortomosaicos adquiridos mediante RPAS para clasificar árboles basados en la presencia binaria de daños por fuego. Las alturas y volúmenes del chamuscado de la corona son luego extractados de árboles dañados por el fuego usando datos de DAP de la nube adquiridos mediante RPAS. Este tipo de análisis para altura y volumen del chamuscado de la corona permite su estimación a través de una escala espacial mucho más amplia de lo que es posible mediante datos de campo.

**Resultados:**

Hay dos diferencias marcadas en los valores espectrales para árboles quemados y no quemados, lo que permite a la metodología desarrollada clasificar correctamente el 92,1% de los árboles como quemados o no quemados. Siguiendo una correcta clasificación, la altura de las coronas chamuscadas de los árboles quemados fueron extraídas con una alta exactitud, que cuando se realizó la regresión con datos de altura medidos a campo dio una pendiente de 0,85, un R^2^ de 0,78 y un valor de RMSE de 2,2 m. Cuando fueron convertidos a volumen de corona chamuscada, el 83,3% de los valores de DAP derivados estuvieron dentro de ± 10% de los valores medidos a campo.

**Conclusiones:**

Esta investigación presenta una metodología post fuego novedosa y costo-efectiva que utiliza datos adquiridos mediante RPAS para caracterizar la severidad del fuego a nivel de árboles individuales a través de una estimación del chamuscado del volumen y altura de la corona. Aunque estos resultados fueron aceptables, algunos mejoramientos pueden ser realizados. Específicamente, a través de la adición de procesos escalonados que podrían remover las sombras y calibrar mejor los datos espectrales usados en este estudio. Adicionalmente, la utilidad de esta aproximación puede hacerse más aparente a través de un detallado análisis de costos, comparando este método con las aproximaciones más convencionales que usan datos de campo.

## Background

The ability to rapidly evaluate the spatial extent and severity of a forest fire is critical to understanding and predicting its short- and long-term impacts on a given ecosystem (Rogan and Franklin 2001; Lentile et al. 2006; Hall et al. 2008; Turner 2010). Conventionally, fire severity mapping is undertaken across broad spatial scales using spectral data acquired from spaceborne satellites (Lentile et al. 2006; French et al. 2008; Szpakowski and Jensen 2019). These methods detect fire-induced spectral changes through the calculation and differencing of pre- and post-fire spectral indices, which are then used to create maps that categorize fire severity across a small number of discrete classes (French et al. 2008). While these methodologies are advantageous due to their extensive use (French et al. 2008), reliance on freely available data (Soverel et al. 2010), and ability to map forest fires over large spatial extents (Hermosilla et al. 2015), they are unable to resolve fire damage that occurs at the individual tree-level, information that is critically important in estimating post-fire tree mortality (Hood et al. 2007; Woolley et al. 2011; Cansler et al. 2020).

There are various ways in which fire damage can be assessed at the individual tree-level, including crown scorch height, which can be expressed as either a height above ground or percentage of damage relative to pre-fire conditions (Alexander et al. 2019). The height above ground at which lethal scorching of foliage occurs is a measure that has long been of interest for fire behavior modelling (Van Wagner 1973) as it is intrinsically linked to fire intensity. A detailed discussion of models that describe these linkages can be found in Alexander and Cruz (2012). Crown damage relative to pre-fire conditions is often more relevant for ecological purposes, as both the percent of crown volume and crown length scorched are commonly used to estimate post-fire tree mortality (Hood et al. 2007; Woolley et al. 2011; Cansler et al. 2020). These post-fire tree mortality models are typically built using extensive datasets that often necessitate the manual measurement of thousands of individual trees (Hood et al. 2007; Woolley et al. 2011), a process that can be expensive and time consuming. Due to practical limitations, these data are commonly collected within individual plots, limiting the types of spatial analysis for which the data can be used. Detailed remote sensing data has the potential to be used to estimate many of the same individual tree measurements, but for every tree within a particular acquisition area.

In recent years, there has been an increased interest in the use of aircraft or remotely piloted aerial systems (RPAS) to acquire imagery at sub-meter resolutions. RPAS-acquired imagery, in particular has seen a rapid uptake across multiple disciples including ecology (Chen et al. 2016; Getzin et al. 2012), forestry (Yancho et al. 2019; Paneque-Gálvez et al. 2014), and fire severity mapping (Fernández-Guisuraga et al. 2018; Larrinaga and Brotons 2019; Arkin et al. 2019). When acquired in a systematic way, RPAS images can also be used to derive digital aerial photogrammetric (DAP) point clouds using structure from motion algorithms. These algorithms match features from overlapping aerial photographs to create three-dimensional point clouds (Carrivick et al. 2016). DAP point clouds have similar applications as those collected using light detection and ranging (LiDAR) methods, with various advantages and disadvantages (White et al. 2013). One such advantage is related to DAP point clouds being constructed from multispectral images (most commonly red, green, and blue bands), resulting in each three-dimensional point containing a spectral response that can be used to detect and characterize photosynthetically active foliage. This type of information is particularly valuable following forest fires, as it can be used to detect living foliage across fire-damaged trees.

In this research note, we examine the capacity of RPAS-acquired orthomosaics and DAP point clouds to assess fire damage across individual trees immediately post-fire. This assessment was carried out by applying a novel two-step method that first uses high spatial-resolution orthomosaics to classify individual trees based on the presence or absence of crown scorch and then assigns a crown scorch height and percent crown length scorched to relevant trees based on the heights and spectral values of DAP points. Approximately 240 ha of RPAS data and 400 field-measured trees were used to validate this novel method.

## Methods

### Study area and data collection

**Fig. 1:**
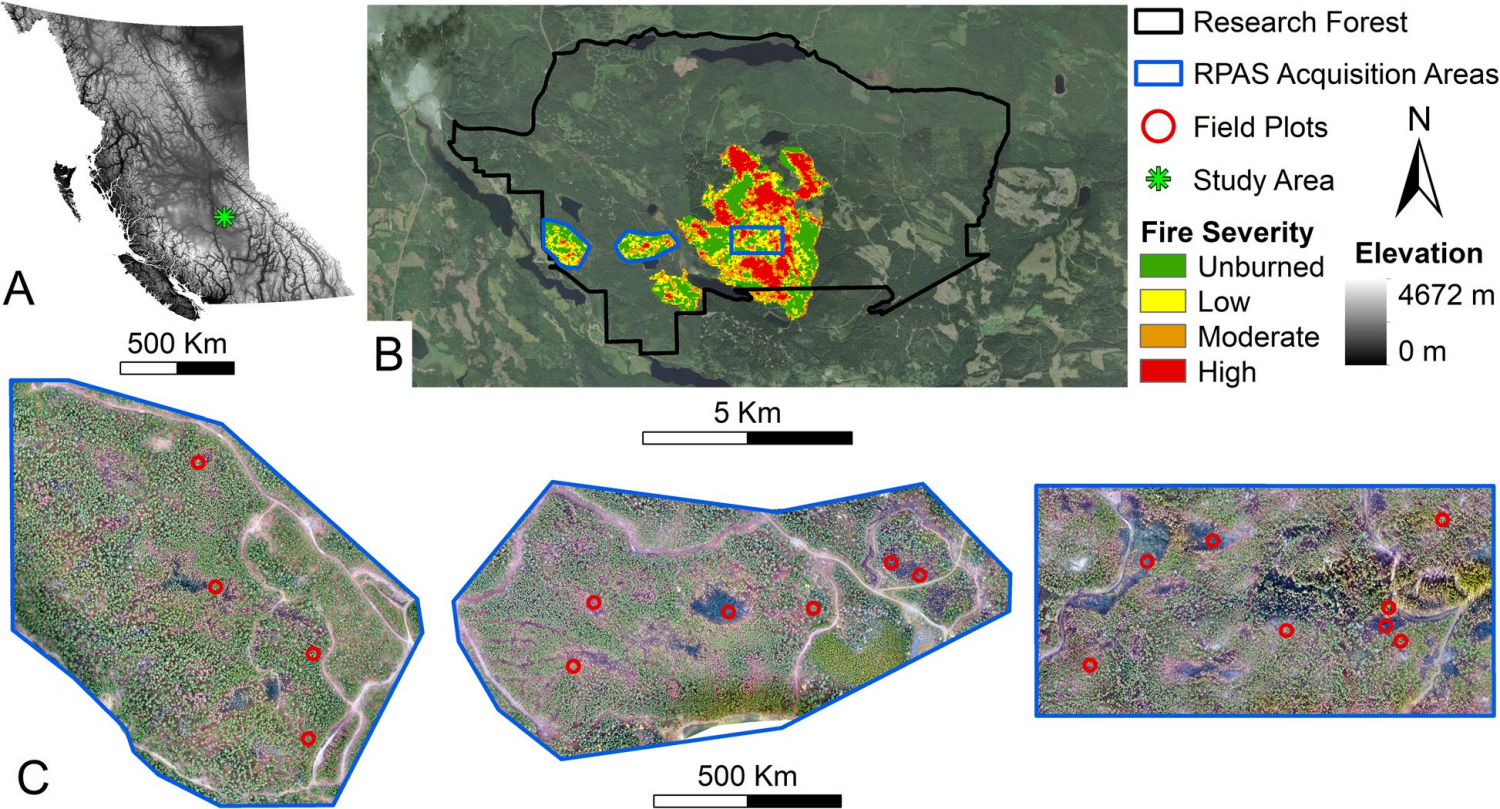
Overview maps of the study area within the Alex Fraser Research Forest. **A** Reference map. **B** Locations of remotely piloted aerial system (RPAS) acquisition areas overlaid on top of a fire severity map classified according to Soverel et al. (2010). **C** Locations of each field plot overlaid on top of RPAS-derived orthomosaics. Background imagery from Maxar

This research was undertaken ~60 km northeast of Williams Lake, British Columbia, Canada, in the northern block of the Alex Fraser Research Forest (Fig. 1), an area categorized as part of the dry warm Sub-boreal Spruce (SBSdw) biogeoclimatic zone (Meidinger and Pojar 1991). During the 2017 fire season, a series of wildfires burned across substantial proportions of the research forest (Prouton Lakes Complex (C30870) (BCWS 2017)). These fires were ignited by lightning in early July and were actively suppressed by ground and air resources until early September, eventually reaching a final area of 859 ha. The final extents and severities of the fires are shown in Fig. 1.

Field and RPAS data were collected from within these fire extents between 22 and 27 October 2017, approximately 1 month after firefighting operations concluded. The RPAS data was collected using an unmodified DJI Phantom 4 over 3 days, with a different acquisition area being collected each day. The acquisition areas averaged ~80 ha in size and included the collection of a series of overlapping aerial photographs that were taken from 120 m above ground level with 90% forward and 85% lateral overlap. To aid in the georeferencing of the RPAS data, 10 ground control points were placed within each acquisition area, for which XYZ locations were collected using either an Ashtech or a Promark3 Global Position System (GPS). The average horizontal and vertical root-mean-square errors (RMSEs) of the GPS points were 1.2 and 1.9 m. More detailed RPAS camera specifications and flight parameters can be found in Arkin et al. (2019).

Field data was collected at eighteen, 11.28-m fixed radius plots, randomly located within these RPAS acquisition areas using a stratified sampling methodology to ensure that a range of fire severities (low, moderate, and high) were equally sampled. Fire severity was classified using relativized differenced Normalized Burn Ratios (RdNBR) derived from pre- and post-fire Landsat scenes according to the methods described by (Soverel et al. 2010).

GPS coordinates were collected at the center of each plot at average horizontal and vertical RMSEs of 1.3 and 1.8 m. Within each of the plots, all trees over 8-cm diameter at breast height (DBH; 1.37 m above ground) were stem-mapped and had a series of measurements taken. Stem-mapping was completed by collecting the distance and azimuth from plot center to each tree bole at breast height using a tripod-mounted TruPulse Laser Rangefinder. Each stem-mapped tree had the following information recorded: species, pre- and post-fire state (living, dying, dead), crown class (dominant, codominant, intermediate, suppressed), DBH, bole char height (lowest and highest), total height, height of live crown, and height of crown scorch. Dead trees were distinguished from dying trees based on the absence of any green foliage.

Crown scorch height was defined as the lowest height of living foliage and later used to calculate crown length scorched. Crown volume scorched was then calculated using the total and scorched crown lengths using the methods described in Hood et al. 2007. Stem-mapped trees were primarily composed of interior Douglas-fir (~75%) (*Pseudotsuga menziesii* var. *glauca*), hybrid white spruce (14%) (*Picea engelmannii* X *Picea glauca*), and lodgepole pine (6%) (*Pinus contorta*), with small components of subalpine fir (*Abies lasiocarpa*), trembling aspen (*Populus tremuloides*), and paper birch (*Betula papyrifera*). Approximately 66% of sampled trees had some amount of visible crown damage, with almost 50% of sampled trees having 100% crown scorch or consumption.

### Data preprocessing

The RPAS data was processed using Agisoft Metashape Pro version 1.7.3 following the same parameters described in Arkin et al. (2019), resulting in the derivation of DAP point clouds at an average density of ~640 points/m^2^ and orthomosaics at an average spatial resolution of 4.41 cm. Following the photogrammetric processing, the generated DAP point clouds were clipped to 30-m radius plot buffers and normalized to ground level using the previously acquired LiDAR point cloud. The normalized point clouds were then clipped to 15-m radius plot buffers and had ground points classified and removed. Points less than 2 m above ground level were also removed. All point cloud processing steps described in this section were completed using LAStools (Isenburg 2017).

### Segmentation and tree matching

Individual tree segmentation was carried out using a marker-controlled watershed algorithm (Meyer and Beucher 1990), which uses a canopy height model (CHM) to segment trees. CHMs were generated for each 15-m radius plot by calculating the maximum point height across 10 cm cells and smoothing the resultant CHM using a Gaussian blur with sigma value of 0.05. Tree tops were extracted from the CHM using a variable local maximum filter that detected all potential trees that were over 2 m in height. The algorithm used the extracted tree tops as seed points to estimate crown boundaries from the smoothed CHMs. In this way, each plot was segmented into many individual tree CHMs, the boundaries of which were used to create and extract individual tree polygons and point clouds.

All segmented trees were visually analyzed and those outside the 11.28-m radius plot or less than 0.3 m^2^ in area were removed. The remaining polygons were matched to field-measured stems. The success of the segmentation routine was evaluated based on the amount of true positive (TP), false negative (FN), and false positive (FP) trees, as well as through the calculation of recall (i.e., sensitivity) $\left(\frac{TP}{TP+ FN}\right)$; and precision (i.e., positive predictive value) $\left(\frac{TP}{TP+ FP}\right)$. All point cloud and CHM-based steps described in this section were carried out using the lidR (Roussel et al. 2020; Roussel and Auty 2021) and ForestTools (Plowright and Roussel 2021) R statistical software (4.0.4) packages.

### Classification of burned and unburned trees

**Fig. 2:**
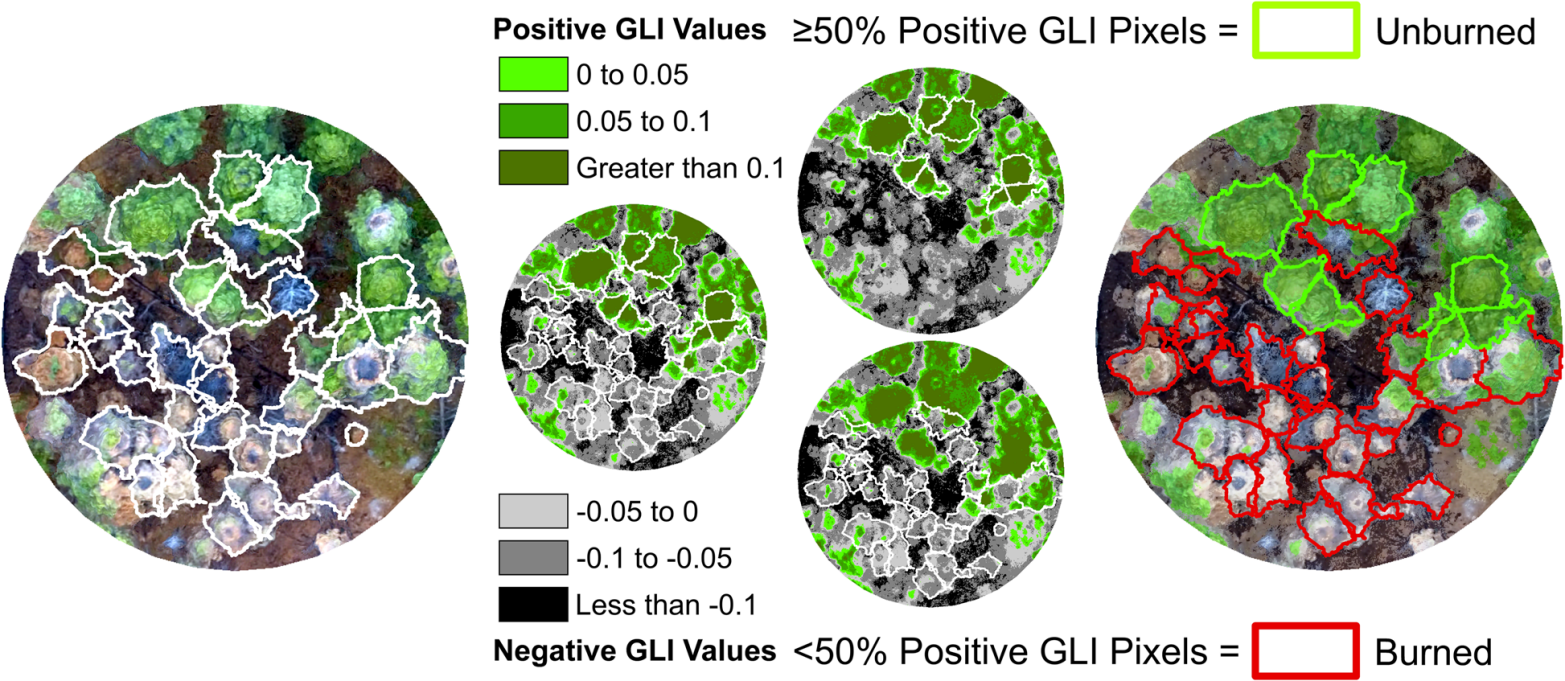
Overview of the classification routine. Following the segmentation of individual trees, Green Leaf Index (GLI) values were calculated for each tree and used to classify each tree as burned or unburned

Due to the presence of shadowed DAP points within both burned and unburned trees, an orthomosaic-based classification was used to classify each TP tree based on the presence or absence of visible fire damage. This classification routine was based on the aggregated greenness values of orthomosaic pixels that were extracted using individual tree polygons (Fig. 2). To ensure pixels were restricted to the area within the crown boundary and to exclude surrounding trees and ground features, pixels were removed if they were below the 25th percentile of tree height, or if they were classified as noise based on an isolated voxels filter (IVF) (Roussel and Auty 2021). The removal of these pixels was necessary due to shadowing effects that were present within the orthomosaics. A detailed concave hull polygon was derived from the remaining points and used to extract orthomosaic pixels for each crown, hereafter referred to as “individual tree orthomosaics”. The greenness of each individual tree orthomosaic was quantified by calculating the Green Leaf Index (GLI) ((2 × *G*) − (*R* − *B*))/((2 × *G*) + (*R* + *B*)) (Louhaichi et al. 2001) value for each orthomosaic pixel. Greenness was summarized at the tree level by calculating the proportion of tree pixels that had positive GLI values. A threshold of 50% was used to classify each tree as either burned (< 50%) or unburned (≥ 50%).

### Estimation of scorch height and crown volume scorched

**Fig. 3:**
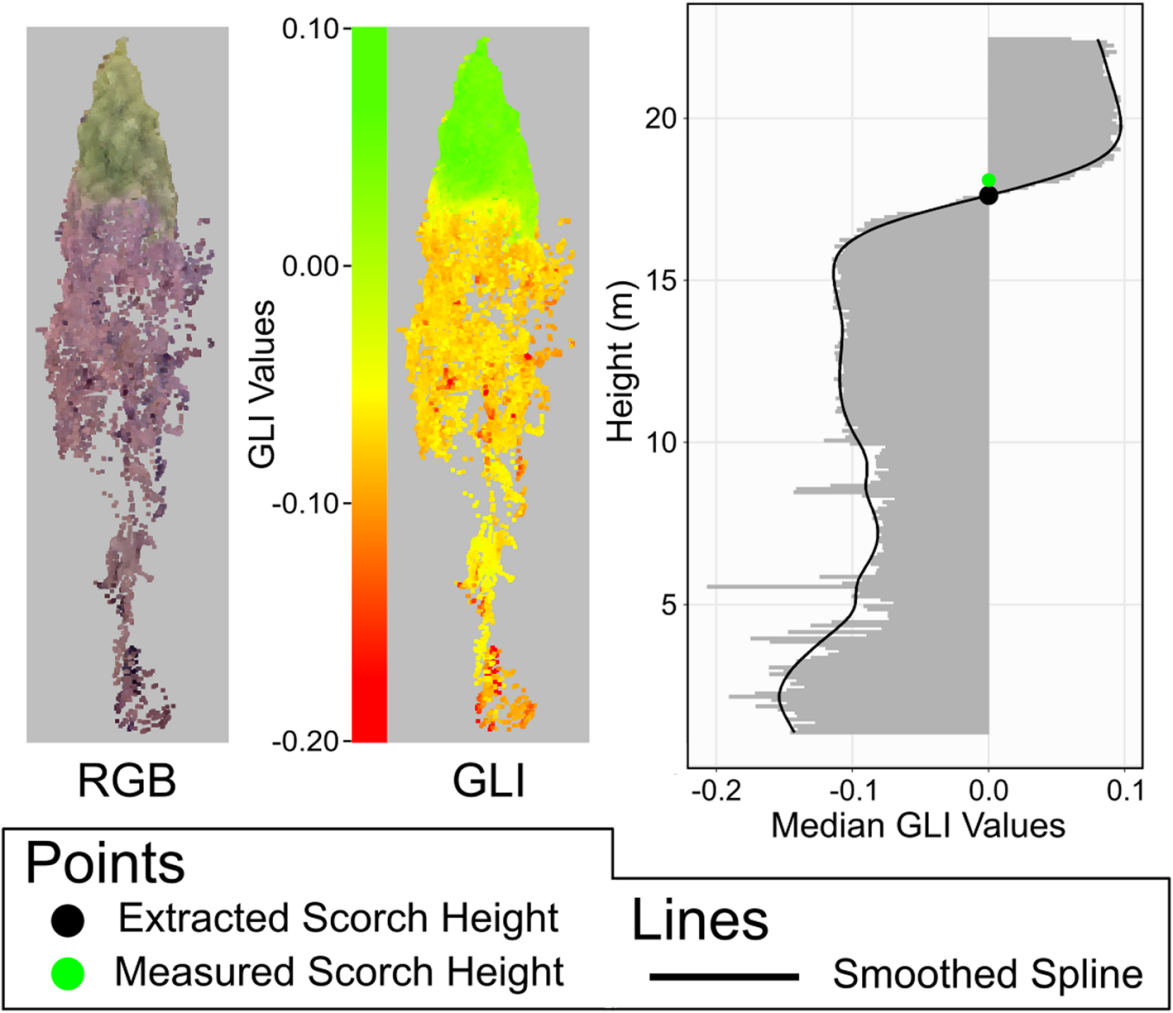
Overview of the scorch height estimation. RGB—red, green, blue; GLI—Green Leaf Index

If a tree crown was classified as burned, crown scorch height and crown volume scorched were estimated using its associated DAP point cloud. To do so, GLI values were calculated for all DAP points, for which aggregated values were vertically analyzed in 10-cm slices of the tree crown (Fig. 3). The height of each slice and its associated median GLI value were used to create a vertical height profile. The trend of the profile was quantified by fitting a spline with a smoothing parameter of 0.65. Scorch height was defined based on the heights at which the fitted spline was negative or positive. Trees characterized by a spline that contained negative and positive values had scorch heights set to the height of the lowest positive portion of the spline. Trees characterized by a spline that was always negative had scorch height set to the maximum point height.

Crown volume scorched (CVS) was estimated using the same methods described in Hood et al. 2007 , using crown length scorched (CLS) and total crown length (CL) $CVS=\left(100\times CL S\right)\left(2\times CL-\left(\frac{CLS}{2\times CL}\right)\right)\times 2$. Crown length required that crown base height be derived for each individually segmented tree, which was accomplished according to the following method. Similar to the estimation of crown scorch height, the derivation of crown base height used vertical profiles from each tree, but in this case using histograms that were built from the number of DAP points within 25-cm vertical slices. Inflection points were then extracted from this profile, with crown base height being defined as the inflection point closest to the ground.

## Results

### Segmentation accuracy

**Fig. 4:**
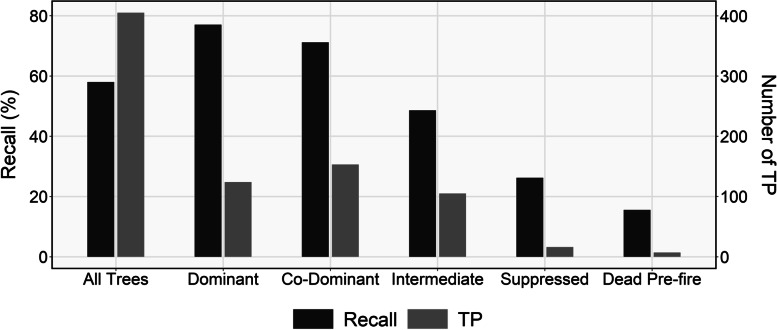
Recall rates and number of true positive (TP) trees by tree class. The TP values refer to the number of trees that were matched to a field-measured tree. Recall rates were calculated by dividing TP by the sum of TP and false negative (FN) values, with FN referring to field-measured trees that were not matched to a segmented tree

In total, 698 trees were measured in the field, of which 405 (TP) were successfully matched to a segmented tree and 293 (FN) were not. One hundred thirty-eight (FP) segmented trees were not matched to any field-measured tree. Respectively, the TP, FN, and FP values resulted in an overall recall of 0.58 and an overall precision of 0.75. The proportion of TP trees and recall rates for each tree class can be seen below in Fig. 4. As expected, both the number of TP trees and the recall rates were highest for dominant (0.77; *n* = 161) and codominant (0.71; *n* = 153) trees, and lowest for trees that were suppressed (0.26; *n* = 61) or dead pre-fire (0.16; *n* = 45). The intermediate trees likely had the largest impact on the relatively poor overall recall due to their abundance in the field data (*n* = 216) and low recall rate (0.49).

### Classification accuracy

**Fig. 5:**
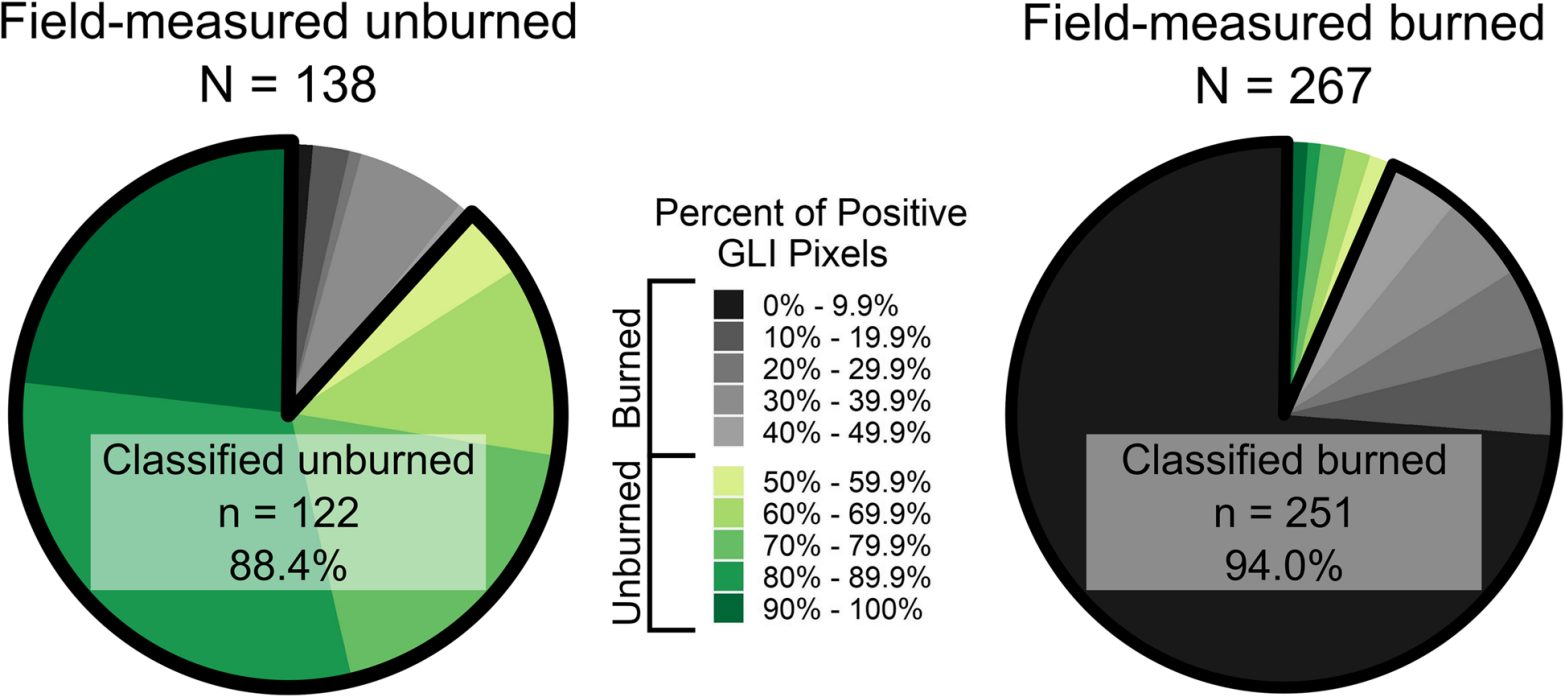
Pie charts showing proportions of positive Green Leaf Index (GLI) values for field-measured unburned and burned trees. Classification accuracies for each group of trees are reported as percentages within each chart

The classification accuracy was favorable for both unburned (88.4%) and burned (94.0%) trees, with an overall accuracy of 92.1% (Fig. 5). In addition to differences in accuracy, the unburned and burned trees had large differences in the relative amounts of positive GLI pixels. The field-measured unburned trees were distinguished by a lack of crown scorch and were characterized by orthomosaics that had a large range of positive GLI proportions, with 85% of the correctly classified trees spanning four classes (60–69.9%, 70–79.9%, 80–89.9%, 90–100%). This is in contrast to the correctly classified field-measured burned trees, of which almost 75% were contained within the class that had fewer than 10% positive GLI pixels.

### Scorch height and crown volume scorched accuracy

**Fig. 6:**
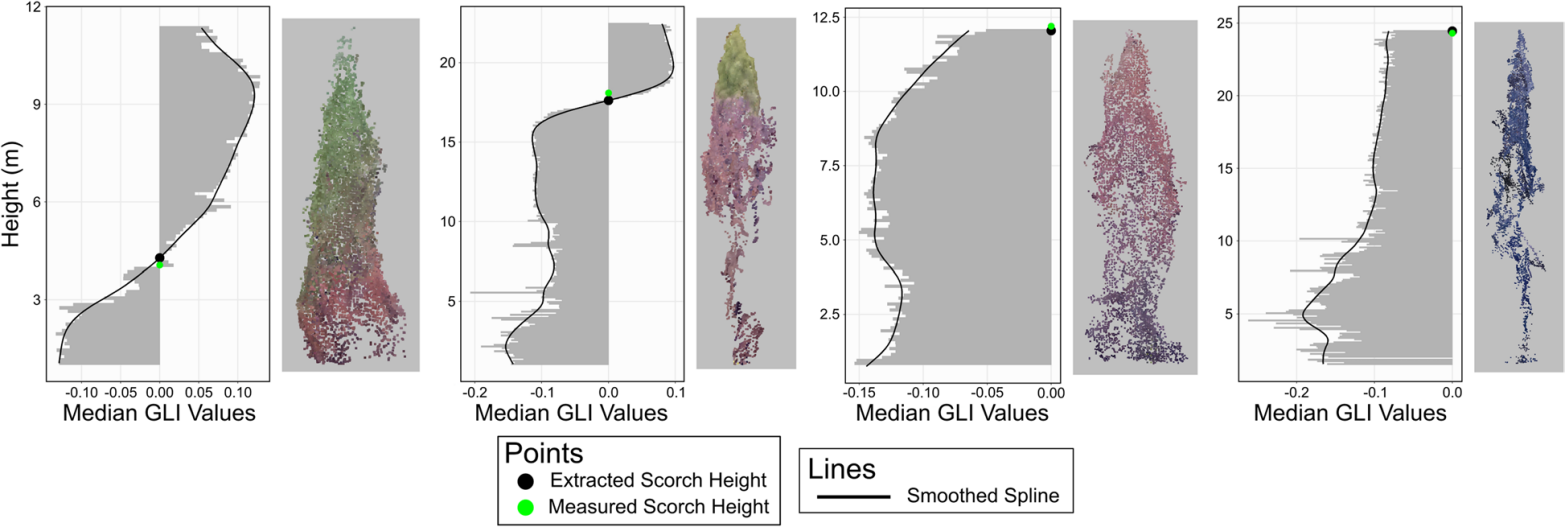
Examples of crown scorch height estimations across four trees showing varying levels of fire damage. The point clouds are shown in true color. Green points correspond to areas of the tree with living needles; red points to areas of the tree with scorched needles; and black points to areas with no remaining needles. GLI—Green Leaf Index

The first two example trees shown in Fig. 6 show clear delineations between scorched and non-scorched portions, with the second having a higher crown scorch height than the first. Both the third and fourth trees had fire damage that extended to the respective tree tops, with the third having 100% crown scorch and the fourth appearing to have had complete crown consumption. In both cases, the scorch height was defined as equal to the maximum tree height.

**Fig. 7:**
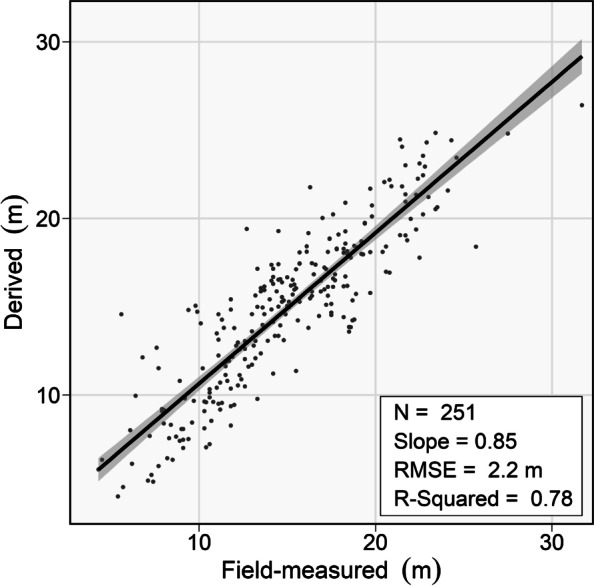
Levels of agreement between field-measured and derived crown scorch heights for trees that were correctly classified as having been burned

When applied to the 251 trees that were correctly classified as burned, the crown scorch height estimation predicted crown scorch heights well (Fig. 7). The strength of this relationship is supported by a slope close to one (0.85), a high R-squared (0.78) value, and a low RMSE (2.2 m) value. The strength of these relationships is relatively consistent between the dominant sampled species. Scorch heights of Douglas-fir trees were estimated with an R-squared value of 0.75, lodgepole pine with a value of 0.79, and hybrid white spruce with a value of 0.86. There were 119 trees that overpredicted crown scorch height by an average of 1.8 m, and 132 trees that underpredicted crown scorch height at an average of 1.7 m. The 16 trees that were incorrectly classified as burned would have had substantial impacts on the strength of the relationship described above (R-squared of 0.36) due primarily to an average estimated scorch height of 17.25 m.

**Fig. 8:**
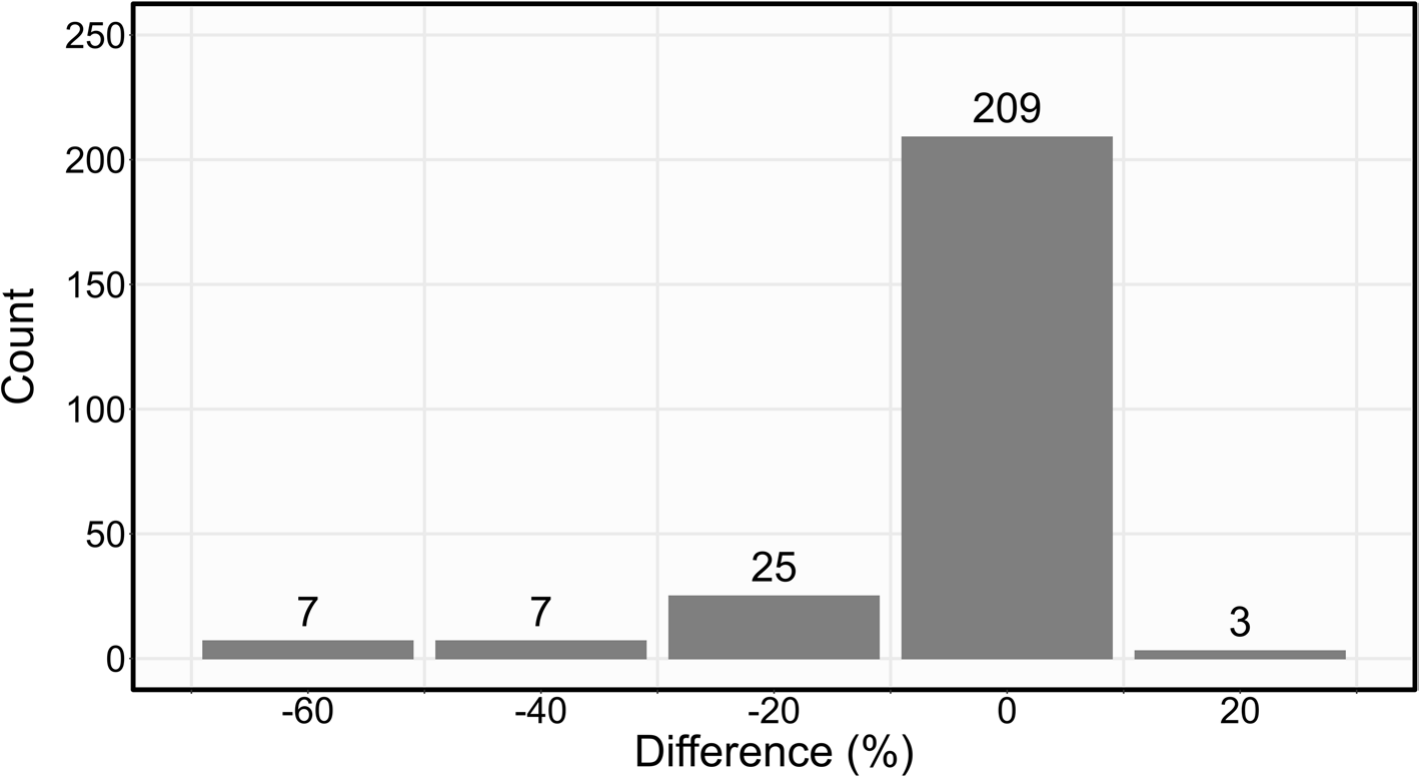
Histogram showing the difference between field-measured and DAP-derived crown volume scorched values. Difference values on the *x*-axis are mid-points of bins spanning ±10%. DAP—Digital Aerial Photogrammetric

Results from the crown volume scorched calculations are shown in Fig. 7. This histogram shows the values derived by subtracting the DAP-derived volumes from the field-measured volumes. A large number (171) of the sampled trees had DAP-derived and field-measured scorch volumes equal to 100%, preventing these results from being displayed in the same manner as Fig. 8. In this distribution, the modal bin is centered around 0, showing that 209, or 83.3%, of the correctly classified trees had a scorch volume estimated within ± 10% of the field-derived scorch volumes. When the error threshold is increased to ±30%, 94.4% of DAP-derived values and field-derived scorch volumes were within this range. The 14 trees with differences of 30% or greater each had much higher values derived from the DAP point clouds than were estimated from the field measurements.

## Discussion

### Classification accuracy

The novel classification routine was able to correctly classify the majority of trees based on a presence or absence of fire damage. The success of this routine was largely due to the more consistent spectral signatures observed for the orthomosaics as opposed to the spectral values in the DAP point cloud. The inconsistency of DAP spectral values were most pronounced in lower portions of tree crowns due to the effect that neighboring trees have on relative illumination (Wallace et al. 2016). Due to these inconsistencies, this method may not be appropriate in dense, multi-layered forests. Additionally, the applicability of this method in non-coniferous forests is unknown, due to their relative absence from the validation dataset. As described above, the misclassification of trees was due to similarities in spectral values between some unburned and burned trees. The misclassified unburned trees all had relatively high proportions of negative GLI values, similar to the proportions observed for many burned trees.

In many cases, the negative GLI pixels in these trees represented bare ground pixels. The amount of bare ground pixels within crown boundaries was reduced based on the point thinning method described in section “Classification of burned and unburned trees”, but some ground pixels remained due to the DAP point clouds’ inability to accurately detect ground features near tree crowns (Graham et al. 2019). Additional negative GLI values were observed with tree crowns of misclassified unburned trees due to variations in crown color among some trees. These variations are likely a result of changes in illumination that, while not as prevalent as in the DAP point clouds, are still common issues within most RPAS-derived orthomosaics (Pons and Padró 2021).

Conversely, the misclassified burned trees all had low proportions of negative GLI values, similar to those of most unburned trees. The relative absence of negative GLI pixels was primarily due to these misclassified trees not having any crown scorch visible from the orthomosaics. Most of these trees had field-measured crown scorch recorded only in lower portions of the crown. These scorched portions were obscured by upper crowns or by dominant neighboring trees that occluded portions of misclassified trees.

### Scorch height and crown volume scorched accuracy

Once a tree was correctly classified as burned, the crown scorch height and volume estimations were highly accurate. Errors in prediction were primarily a result of differences in how the field data were collected compared to how the segmented DAP point clouds were analyzed. The field measurements recorded the lowest crown scorch height on a particular tree, whereas the point clouds required a majority of DAP points to have negative values within a 10-cm slice of a tree. In cases where the crown was scorched along a horizontal line, these differing methodologies resulted in nearly identical measures. These differences would have been reduced, but not eliminated, if crown scorch height was estimated as the highest point of scorched foliage. The largest differences in extracted and measured heights occurred where the crown was scorched more on one side of the tree than the other. In these cases, the field data suggested a much lower crown scorch height than was derived from the DAP point cloud. These differences were particularly pronounced in the crown volume scorched results, with 14 trees having scorched volumes estimated between 30 and 64% higher than the field-measured values.

While 698 trees were sampled in the field, only 405 were successively matched to a DAP-derived tree. Of these 171 had crown scorch volumes equal to 100%, and another 138 were unburned. This only left 96 trees that had scorch volumes between 0 and 100%. As these trees are the most difficult to characterize, future studies should seek to selectively sample a higher proportion of trees that had moderate crown scorch volumes. Additionally, field measurements that more directly quantify the percent of needles that were scorched may have revealed more nuanced results that could further inform this methodology. Crown volume scorched was estimated from total crown length and scorched crown length (Hood et al. 2007); however, we acknowledge this method works best for trees that have uniform crown scorch heights and fails to accurately estimate scorched volume when one side of the crown has a higher scorch height than the other. Although more time consuming, a more detailed measurement of three-dimensional crown scorching patterns in the field would have been valuable. These measurements could then be used to validate a more direct estimation of crown scorch using DAP point clouds. Additionally, while not included in this analysis, this work suggests that similar methods could be used to distinguish between portions of the crown that were scorched versus consumed, measurements that have been improved the accuracy when modelling delayed post-fire tree mortality (Fowler et al. 2010; Varner et al. 2021). Such methods would further support a more detailed, three-dimensional measuring of fire damage patterns in the field.

### Limitations and future work

The ability of DAP point clouds to record spectral information in addition to structural information represents a key strength of the technology. However, the application of DAP is fundamentally limited by its inability to derive subcanopy points to the same degree as LiDAR (White et al. 2013). As shown by the relatively low recall rates, this weakness limits the number of intermediate and suppressed trees that can be analyzed by this method. A similar lack of DAP points was observed in areas that burned at the highest severities, especially within areas where tree crowns were completely consumed by the fire. Two plots used for this study characterized such areas, in which only ~12% of field-measured trees had DAP points derived. While the lack of derived trees can be simply attributed to complete crown consumption, this is still a limitation. The derivation of DAP points that can reliably reconstruct subcanopy trees and individual tree boles could have been improved through the collection of oblique RPAS images (Perroy et al. 2017; Ye et al. 2019; Moreira et al. 2021). Such images could have supplemented the NADIR RPAS images used in this study. The ability to reliably characterize tree boles could allow for the extraction of bole char heights, another critical post-fire metric (Woolley et al. 2011), using similar methods as presented here.

The RPAS images used to derive the orthomosaics and DAP point clouds were collected around solar noon under optimum light conditions in order to ensure the spectral quality of the data. Despite these precautions, there was still considerable shadowing present within the DAP point clouds and, to a lesser degree, the RPAS-derived orthomosaics, especially for more obscured features. As previously mentioned, this shadowing likely contributed to the amount of negative GLI values within unburned crowns. Radiometrically calibrating these data would likely have minimized these unexpected negative GLI values and is increasingly being seen as a necessary processing step within any image-based RPAS methodology (Assmann et al. 2019; Pons and Padró 2021). These issues could be further mitigated by collecting RPAS images that contain non-visible bands. Red-edge and near infrared bands are particularly advantageous as they allow for the derivation of spectral indices that are more sensitive to vegetation than their visible band counterparts (Carvajal-Ramírez et al. 2019). The inclusion of these non-visible bands in conjunction with a radiometric calibration step would likely increase the overall accuracy of the approach presented in this study.

The focus of this research is on the development and use of RPAS-based remote sensing methods to estimate crown scorch heights and volumes within individual conifer trees. This type of analysis has value in estimating fire intensity (Van Wagner 1973), predicting delayed mortality (Woolley et al. 2011), and ultimately identifying short- and long-term fire refugia (Downing et al. 2019). These values notwithstanding, there are certain limitations associated with RPAS-acquired data. In addition to those discussed above, the short flight times associated with most RPAS limit the ability to apply these methods across large areas. Tradeoffs between spatial coverage and spatial resolution are common in the application of any remote sensing system. In this way, RPAS data should be considered as one part of a multi-scale continuum of post-fire remote sensing tools that decrease in spatial resolution as they increase in spatial extent. RPAS-acquired data are well suited to analyze fire impacts at the finest levels of detail, but only over relatively small areas (McKenna et al. 2017; Larrinaga and Brotons 2019; Arkin et al. 2019). Conversely, most satellite acquired data are well suited to analyze fire impacts over extremely large areas, but at more coarse levels of detail (Lentile et al. 2006; French et al. 2008; Hermosilla et al. 2015). The development of a more integrated framework would allow for RPAS data to be used to supplement conventional, satellite-based fire severity maps with more detailed information in areas of highest concern. In order to support this framework, a thorough review should be carried out in order to compare the cost, scale, and accuracy of post-fire measurements across a continuum of platforms.

## Conclusion

The novel methods presented in this research successfully analyzed fire impacts across individual trees segmented from DAP point clouds. A large majority of trees were correctly classified using an orthomosaic-based approach, including 88.4% of unburned trees and 94.0% of burned trees. For successfully classified burned trees, crown scorch heights and volumes were reliably estimated. Field-measured and extracted crown scorch height values corresponded well, with an R-squared value of 0.78 and an RMSE value of 2.2 m. Crown volume scorched estimates were also carried out very accurately, with 83.3% of DAP-derived values being with ±10% of field-derived values. These favorable results indicate that this method can reliably assess and map crown scorch height across large numbers of individually segmented trees. Such estimations of crown scorch height have value in estimating maximum fire intensity, predicting delayed mortality, and identifying tree refugia. While the potential application of these methods is promising, there are certain limitations associated with the data and methods used in this study. Primary among these is related to an inability to reliably detect and characterize understory trees from the RPAS-acquired orthomosaics and DAP point clouds. The methods used in this study also did not include any additional steps to radiometrically calibrate or to remove shadows from the data. Such steps would likely increase the accuracy and reliability of these methods. We recommend the future inclusion of acquisition and processing steps to address these limitations. Additionally, we recommend the development of a more integrated remote sensing framework that would allow for fire severity to be analyzed and mapped using systems of various spatial scales and resolutions depending on particular management needs.

## Data Availability

Software code and all data are available upon request from the corresponding author.
